# Attending to cross-border sociocultural competence in bilingual programs in the Polish-Czech border region: An exploratory study

**DOI:** 10.1371/journal.pone.0293069

**Published:** 2023-11-30

**Authors:** Megan Hopkins, Joanna Kurowska-Pysz, Edyta Nowak-Żółty, Michał Szyszka

**Affiliations:** 1 Department of Education Studies, University of California, San Diego, San Diego, California, United States of America; 2 Faculty of Applied Sciences, WSB University, Dabrowa Gornicza, Poland; The University of Auckland, NEW ZEALAND

## Abstract

The aim of this research was to explore whether and how various components of cross-border sociocultural competence were attended to in bilingual schools serving Polish minorities in the Zaolzie border region. Zaolzie is on the Czech side of the Polish-Czech border region, where the Polish minority play an important role in cross-border relations. Based on a review of the extant literature on the development of sociocultural competence among students enrolled in bilingual programs, researchers developed a 19-item survey capturing three distinct components of cross-border sociocultural competence: cognitive, social, and political. The survey was sent to teachers in the 14 Zaolzie region schools, with 123 teachers (44%) participating and asked participants to indicate the extent to which different facets of cross-border sociocultural competence were part of their school’s instructional program. Most participants were female-identifying and had over 10 years of teaching experience, and most were born on the Czech side of the border but identified as Polish nationals. Results from an exploratory factor analysis (EFA) revealed two unique factors representing different components of sociocultural competence included in bilingual programs in the Zaolzie region: (1) the development of students’ intrapersonal skills, motivation, and cross-linguistic understanding, and (2) the development of students’ capacity to engage interpersonally with and to understand others in the region. These findings suggest that schools in the Zaolzie region attend to cross-border sociocultural competence through both an intra- and interpersonal lens, emphasizing individual students’ linguistic and cultural development as well as their ability to interact in their highly politicized cross-border context. Educators in the region can thus consider assessing students’ progress in these two areas over time as measures of program effectiveness. Given that the researchers found that cognitive, social, and political aspects of sociocultural competence were integrated across intra- and interpersonal dimensions, more research is needed to understand if this integration is unique to the teaching of cross-border sociocultural competence in the Zaolzie region or is taught similarly in other contexts.

## Introduction

The economic and social importance of border regions has proliferated over the last few decades, stemming from broader patterns of globalization “when the movement of people, goods, or ideas among countries and regions accelerates” [1]. The growth of cross-border economic activities, the “territorialization of political power” through regional integration, and the integration of socialist economies into global capitalism at the end of the Cold War increased the permeability of borders and opportunities for economic transformation in border regions [2]. Although globalization and cross-border cooperation have received a great deal of attention in the fields of economics and political science, less emphasis has been given to how accelerating cross-border dynamics are shaping, and are shaped by, education, and particularly elementary and secondary education [3].

The transformations occurring in border regions around the world require children and youth to develop new skills to be informed, engaged, and critical members of the global community. Some scholars in the field of education refer to these skills as *sociocultural competence*, which can enable students’ engagement in and with diverse cultural communities [4, 5]. The concept “begins with an understanding of how students are coming to understand and view themselves” as bilingual and bicultural individuals [5] and extends to understanding how their identities and those of others are situated “within particular histories of power” [6]. As something that must be deliberately taught and developed over time, the teaching of sociocultural competence can support students’ positive identity development and self-esteem as well as their capacity to embrace difference, value diversity, and strive to address societal inequalities.

While teaching sociocultural competence is an espoused goal of many bilingual and dual language education programs, especially in the United States [4], it is an essential component of elementary and secondary education in border regions, where diverse narratives of communities living on either side of the border overlap, as national and ethnic minorities live, work, and attend school on both sides of the border [7]. As a potential foundation for developing students’ sense of belonging to communities on both sides of the border, attention to cross-border sociocultural competence in elementary and secondary education may be important for facilitating cross-border cooperation and the development of multidimensional cross-border socio-economic ties [8].

In this paper, we present findings from a study examining whether and how various components of cross-border sociocultural competence are attended to in bilingual schools serving Polish minorities in the Zaolzie border region. Historically defined by social tensions stemming from previous bilateral military aggressions, Zaolzie Polish minorities co-exist with the Czech majority in this cross-border region and are interconnected socially, economically, and politically. Schools thus represent an important site in Zaolzie for mitigating any remaining tensions, as they can explicitly address linguistic and cultural differences that may shape students’ daily lives and long-term trajectories [9].

In the sections that follow, we begin by further motivating our attention to the development of students’ border-specific sociocultural competence; then, we outline three components of sociocultural competence that have been identified in the extant literature in the field of education. After that, we describe our methods for survey data collection and analysis. Next, we present our findings related to the two unique factors identified through our analysis and discuss their implications for educational practice and research in cross-border regions and beyond.

### The importance of border-specific sociocultural competence

We understand sociocultural competence as foundational for productive work, generative play, and meaningful co-existence amongst diverse languages and cultures. Border regions are unique contexts in which two or more languages and cultures intertwine, and there is an economic and social need for inhabitants of both sides of the border to learn from, and with, one another as the result of complex geopolitical processes. Throughout history, there have been many changes to borders. With these changes, the intermixing of different nationalities, cultures, languages, religions, and identities, has led to the gradual development of distinctly diverse border communities [10, 11]. The integration of cultures and the development of various types of cross-border relationships (e.g., interpersonal, social, and economic) provides opportunities for members of border communities to develop border-specific values, customs, traditions, and social norms. These processes can lead to the development of a unique identity within the border region based on factors including territorial proximity, language, religion, ethnic community, history, and tradition [12].

Border regions often comprise various social groups that differ along linguistic, ethnic, religious, or national lines. Many of these groups include minorities who bond through a shared history and cultural and linguistic practices [13]. At the same time, minority groups are often under pressure from the dominant group to assimilate [14]. Reconciling this tension in some border regions can be a great challenge, given that interactions between minorities and the dominant group often emphasize the differences of interests, needs, and aspirations that separate them rather than foregrounding their emerging multicultural identities [15]. Although cultural identity arises from a conscious orientation to the values of the group or groups with which an individual identifies [16–18], membership in one group does not exclude participation in or cooperation with other groups [19]. Simultaneous identification with various groups, which occurs naturally in cross-border regions, enables the formation of a multicultural identity [20]. This identity is unique to the border regions where those various cultural and linguistic groups interact in their personal and professional lives.

The complex and fluid identities exhibited by inhabitants of cross-border communities suggest that unique sociocultural competences are necessary to engage in contexts where cultures and languages intertwine. Border-specific sociocultural competence facilitates bilingual and bicultural interactions in an area where two or more languages and cultures intertwine and where there is an economic and social need to learn from, and with, one another as the result of complex geopolitical processes [11, 12]. The development of such competencies allows communities living in border regions to take full advantage of the opportunities offered by life on both sides of the border, opportunities that are essential for the development of cross-border cooperation and for counteracting intergroup tensions arising from the marginalization of certain racial, ethnic, linguistic, and cultural groups [16]. Attending to sociocultural competence may thus help to facilitate interregional and international relations that enable territorial cooperation.

The uniqueness of border regions as a sociocultural phenomenon, where many political, historical, ethnic, religious, and other dimensions intersect, suggests the need for education systems to focus on the development of cross-border sociocultural competence for children and youth living in border regions. Indeed, education plays a key role in the development of sociocultural competence, with respect to shaping students’ knowledge, skills, attitudes, and awareness [5, 21] that will prepare them for life, work, and leisure, as well as building professional, social, and personal relationships within their cross-border context. Our study seeks to examine whether and how different components of cross-border sociocultural competence are included in the instructional program of a specific set of schools in the Polish-Czech border region. To delineate these components, we turn to the literature on second language learning and bilingual educational programs, as described next.

### Components of teaching sociocultural competence

Scholars studying peace and conflict resolution have for many decades emphasized the importance of intercultural competence for individuals, groups, and organizations as they engage in diverse cultural contexts [22–25]. Assessing intercultural competence can support individuals with identifying their areas of strength and opportunities for growth and can help groups and organizations achieve diversity and inclusion goals [26]. These studies have generated a range of instruments for assessing intercultural competence in areas like cultural awareness, attitudes about diversity, and communication strategies [27]. These areas have also been identified as important in the literature on second language teaching and learning, which emphasizes the need to specifically attend to the interdependence of culture and language in defining one’s individual identity as well as one’s engagement with others across lines of difference [28–30]. Through educational experiences such as study abroad, students can develop the ability to see relationships between different cultures, understand one’s own culture and other cultures, and engage in intercultural communication [31].

Teaching sociocultural competence has also become a prominent theme in research on dual language programs in the United States. Dual language programs are bilingual programs offered in primary and secondary schools to provide instruction to students in two languages, with the goal of: “promot[ing] bilingualism and biliteracy, grade-level academic achievement, and sociocultural competence—a term encompassing identity development, cross-cultural competence, and multicultural appreciation—for all students” [4, p. 3]. In line with this definition, research examining the teaching of sociocultural competence in dual language programs tends to emphasize the benefits of these programs on students’ attitudes toward themselves *and* on their attitudes toward other languages and speakers of those languages [5, 32, 33]. These attitudes are often considered separately in examinations of dual language program outcomes, as having a strong sense of one’s identity is often separate from, and even a prerequisite to, developing robust cross-cultural attitudes and intercultural communication skills [5, 30, 34]. Thus, instruction in dual language and other bilingual programs often focuses on valuing and honoring students’ home languages and cultures to promote positive identity development as well as providing opportunities for students to develop bilingual and bicultural skills to support communication in diverse contexts [35].

Beyond individual identity development and intercultural communication skills, recent scholarship examining student interactions in dual language programs exp suggests that facets of sociocultural competence are mediated by the inequalities that tend to exist between linguistic and cultural groups [36, 37]. These inequalities influence students’ learning experiences in dual language programs as societal power dynamics play out in their daily classroom interactions [6, 38, 39]. Thus, beyond individual student’s identities and cross-cultural attitudes, it is important to attend to students’ developing awareness of the status of languages in their community. Often referred to as *critical consciousness*, this facet of sociocultural competence can help students understand how their language learning is influenced by societal inequalities and how they can attend to these inequalities as they interact in their communities [6].

Based on this literature, we articulated three components of sociocultural competence that are taught in bilingual programs: 1) a cognitive component focused on individual skill and identity development; 2) a social component focused on cross-cultural awareness and intercultural communication skills; and 3) a political component focused on developing critical consciousness (see Table 1).

**Table 1:** Components of sociocultural competence.

Component	Definition
Cognitive (i.e., bilingual/ bicultural identity	Students develop bilingualism and biliteracy and have positive associations with being bilingual and bicultural as evidenced through enhanced self-esteem and motivation to learn two languages
Social (i.e., cross-cultural competency)	Students hold positive attitudes and empathy toward different linguistic and cultural groups and display flexibility in their ability to engage within and across different languages and cultures
Political (i.e., critical consciousness)	Students demonstrate awareness of language status, power dynamics, and related inequalities between linguistic and cultural groups and express value for the preservation of family and community ways of knowing

## Methods

Considering that this research aimed to explore whether and how various components of cross-border sociocultural competence were attended to in bilingual schools serving Polish minorities in the Zaolzie border region, we pointed out two research questions:

1. What are the components of cross-border sociocultural competence in bilingual schools serving Polish minorities in the Zaolzie border region?
2. What items are essential in individual components of cross-border competence in these schools?

**Fig 1:**
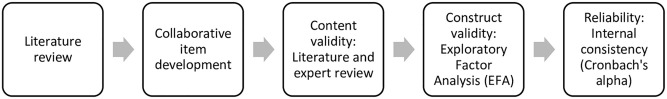
Phases of survey development and analysis.

We developed a school staff survey to examine whether and how bilingual schools in the Zaolzie region attend to various components of border-specific sociocultural competency. We surveyed teachers in the 14 schools serving Polish minorities in the region and conducted an exploratory factor analysis to determine the underlying components that constitute sociocultural competency in this specific border context. The phases of survey development and analysis are described below and displayed in Fig 1.

### Instrument development

After identifying the three overarching components of sociocultural competence from the literature (see Table 1), we engaged in a collaborative process to ensure that survey items pertaining to each component would be understandable and relevant to teachers in schools in the Zaolzie region. Given that existing surveys focus primarily on assessing individual and organizational sociocultural competence rather than on what aspects of sociocultural competence are taught to students [27, 34], our international research team drafted original survey items. These items asked teachers how important various facets of cross-border sociocultural competence are to the goals of their school, with items rated on a five-point Likert scale range from 1 (*Unimportant*) to 5 (*Very important*).

To ensure a border-specific emphasis, we considered how the schools’ immersion in a highly fluid border region might add complexity to each of the three components identified in the literature. For example, we leveraged our own review of the cross-border literature as well as a recent review of students’ experiences in US border communities [40] to incorporate the unique sociocultural, sociolinguistic, and sociopolitical facets of schooling in cross-border contexts. Content validity was also supported through an expert review of the items by researchers and key community stakeholders from the Zaolzie region. All three experts are bilingual, have advanced degrees in education, and previously taught in Polish schools. They also conduct studies on bilingual education. They represented the key organizations and institutions responsible for supervising bilingual and bicultural education in the Zaolzie region, including: the Pedagogical Center for Polish National Education in Český Těsín established by the Czech Ministry of Education (the Czech Republic); the Congress of Poles in the Czech Republic; and the Polish Cultural and Educational Association in the Czech Republic. The experts assessed the readability of the questionnaire and the relevance of the questions to teachers in the schools in the Zaolzie region. They also noted issues with wording and some translations and recommended some changes in the language of several items.

The resulting survey included 19 items addressing sociocultural competence: six items pertaining to the cognitive component, eight items related to the social component, and five items related to the political component. These 19 items, and the component to which each aligns, are indicated in Table 2.

**Table 2:** Survey items and their associated components.

How important are the following goals at your school?	Component of Cross-Border Sociocultural Competence		
	Cognitive	Social	Political
1. Develop students’ bilingualism and biliteracy	X		
2. Develop students’ proficiency in the dialect of the border region	X		
3. Promote students’ multilingual and multicultural identities	X		
4. Promote students’ motivation to learn multiple languages and cultures	X		
5. Support positive perceptions of students’ and families’ languages and cultures	X		
6. Develop students’ understandings of language similarities and differences	X		
7. Develop students’ awareness of the differences between cultures and traditions of the neighbouring nations		X	
8. Develop students’ empathy and understanding for different points of view		X	
9. Develop students’ ability to engage in various cross-border relations		X	
10. Promote a sense of Polish-Czech togetherness on the border		X	
11. Promote respect for majorities and minorities equally		X	
12. Promote curiosity, tolerance and openness for other cultures, traditions, behaviour norms and styles of thinking		X	
13. Develop students’ appreciation for the cultures and traditions represented in the border region		X	
14. Develop students’ abilities to use different language(s) in different situations as appropriate		X	
15. Develop students’ historical understanding of the border region			X
16. Develop students’ awareness of minority rights			X
17. Develop students’ awareness of characteristics of the border region in terms of both professional and private life			X
18. Support families to maintain the heritage of the language and traditions of the ancestors			X
19. Support awareness of differences in language status in the border region			X

*Note*. Although the items are grouped by component in the table, they were asked in a different order on the survey to intersperse the items related to each component.

### Data collection

This study was conducted as part of the project “Regional Initiative of Excellence” in 2019–2023, project number 018/RID/2018/19, carried out at WSB University in Dąbrowa Górnicza, Poland. The study approval from the departmental research committee and was conducted while maintaining research ethics in the social sciences. After receiving research approval, an online survey was sent in September 2022 to all teachers in the 14 Polish-Czech bilingual schools in the Zaolzie region. Teachers’ participation was voluntary, and survey respondents could opt out of completing the survey at any stage. Participants were informed about the purpose of the research, as well as about the ways in which the data–the answers provided–would be processed. The research tool did not include questions that would personally identify any participants.

### Sample

A total of 123 teachers participated in the survey across the 14 schools, representing 44% of all teachers in these schools. Most of the sample was female-identifying and had more than 10 years of teaching experience. Whereas most teachers were born on the Czech side of the border, they identified as Polish nationals (see Table 3).

**Table 3:** Participant demographics.

	Female-identifying	Teaching experience				Place of birth		Nationality	
		0–5 years	6–10 years	11–20 years	21+ years	Polish side	Czech side	Polish	Czech
Percent of sample (n = 123)	84%	15%	19%	33%	33%	8%	88%	95%	3%

### Data analysis

We conducted an exploratory factor analysis (EFA) to identify the structure of the underlying data and reveal the underlying constructs comprising cross-border sociocultural competence. To support construction validity, we conducted a maximum likelihood EFA and examined goodness-of-fit statistics to determine the compatibility of our theorized three-component model with the data [41]. Varimax rotation was used to maximize distance between the factors because of the likelihood that they would be correlated [42]. Items with a factor loading higher than .5 on the primary factor and lower than .3 on the secondary factor were noted as consistent with the factor. Items that loaded higher than .3 on the secondary factor (or split .4/.2) were noted as multidimensional and in need of additional analysis [42].

Model fit to the data was determined using structural equation modelling to generate the following goodness-of-fit statistics: For the comparative fit index (CFI) and the Tucker–Lewis index (TLI), values above .90 indicate adequate fit [43]; for the root-mean-square error of approximation (RMSEA), .06 or below indicates adequate fit; and for the standardized root mean-square residual (SRMR), .08 or below indicates adequate fit [44]. We report the Chi-square statistic (v2); however, because Chi-square is sensitive to model complexity, assumption violation, and sample size, we depend on the foregoing parameters to determine model fit [41].

As will be described below, we used this analytical approach for both a three-factor and two-factor solution, given that our initially theorized three-component structure did not produce favourable results. After completing these analyses, we calculated Cronbach’s alpha for the resulting two factors to examine their internal consistency.

## Results and discussion

### The components of cross-border sociocultural competence in bilingual schools serving Polish minorities in the Zaolzie border region

The mean and standard deviation for each of the 19 items is displayed in Table 4. The highest mean (4.47) was observed for the item: “Develop students’ abilities to use different language(s) in different situations as appropriate,” while the lowest mean was observed for the item: “Develop students’ proficiency in the dialect of the border region” (3.78). Teachers thus tended to report that each of the statements was at least moderately important to their school’s instructional program. Standard deviations ranged from 0.73 to 1.02, indicating at most a one-point variation in teachers’ ratings of importance on some items (e.g., the difference between marking “very important” and “moderately important”). Skewness and kurtosis values were all within a reasonable range (all items below an absolute value of 1.96).

**Table 4:** Survey items and their associated components.

Item	Descriptive statistics		Factor loadings on each component			Included in factor 1, 2, or none
	Mean	SD	1	2	3	
1. Develop students’ bilingualism and biliteracy	4.46	0.91	0.52			1
2. Develop students’ proficiency in the dialect of the border region	3.78	1.01		0.34		None
3. Promote students’ multilingual and multicultural identities	4.14	0.73	0.42	0.32		None
4. Promote students’ motivation to learn multiple languages and cultures	4.39	0.91	0.78			1
5. Support positive perceptions of students’ and families’ languages and cultures	4.28	0.86	0.66			1
6. Develop students’ understandings of language similarities and differences	4.15	0.95	0.49			1
7. Develop students’ awareness of the differences between cultures and traditions of the neighbouring nations	4.15	0.76	0.53			1
8. Develop students’ empathy and understanding for different points of view	4.22	0.74	0.36	0.63		2
9. Develop students’ ability to engage in various cross-border relations	3.84	0.86		0.55		2
10. Promote a sense of Polish-Czech togetherness on the border	4.04	0.74		0.63		2
11. Promote respect for majorities and minorities equally	4.18	0.83		0.66		2
12. Promote curiosity, tolerance and openness for other cultures, traditions, behaviour norms and styles of thinking	4.24	0.93	0.34	0.42		None
13. Develop students’ appreciation for the cultures and traditions represented in the border region	4.04	0.92	0.52			1
14. Develop students’ abilities to use different language(s) in different situations as appropriate	4.47	0.86	0.56			1
15. Develop students’ historical understanding of the border region	4.36	0.83	0.42	0.41		None
16. Develop students’ awareness of minority rights	4.13	0.81			1.23	
17. Develop students’ awareness of characteristics of the border region in terms of both professional and private life	3.79	0.86		0.40		None
18. Support families to maintain the heritage of the language and traditions of the ancestors	4.27	1.02	0.30	0.59		2
19. Support awareness of differences in language status in the border region	4.25	0.83	0.52	0.41		1

Initial EFA results pointed to a potential two-factor solution versus a three-factor solution, as Factor One had an eigenvalue of 6.45 and accounted for 73% of the variance in sociocultural competence, and Factor Two had an eigenvalue of 1.42 and accounted for an additional 16% of the variance. Factor Three had an eigenvalue below one (0.93) and accounted for another 11% of the variance. Table 4 displays pattern factor coefficients above 0.3 demonstrated through the EFA, further revealing a two-factor solution, as only one item (“Develop students’ awareness of minority rights”) loaded on the third factor. For the items that loaded on both Factor One and Factor Two (#3, 8, 12, 15, 18, and 19), if the factor coefficient was greater than 0.5 and made theoretical sense to include with the other items loading on that factor, we selected the factor accordingly. If, however, the factor loadings were both less than 0.5 (#3, 12, and 15), then we included them in additional analyses as described below.

We examined goodness-of-fit statistics for our originally theorized three-factor solution, which as expected given our initial EFA results did not suggest a good fit (see Table 5). We thus proceeded with constructing a two-factor solution and refined item inclusion within each. For example, item #15, “Develop students’ historical understanding of the border region,” loaded nearly equivalently onto both factors; thus, we included it sequentially in each factor and compared the goodness-of-fit statistics to determine the best fit to the data. If the fit was best without including the item, then the item was dropped. The final two factors and their associated items are indicated in Table 4 and the goodness-of-fit statistics for the best fitting two-factor solution are shown in Table 5.

**Table 5:** EFA goodness-of-fit indices for two- and three-factor models.

Model	Chi-square	Df	P value	RMSEA	CFI	TLI	SRMR
Three-factor	871.33	171	0.00	0.08	0.84	0.82	0.08
Two-factor	568.65	91	0.00	0.05	0.96	0.96	0.06

Considering this, our results identified two factors representing distinct dimensions of border-specific sociocultural competence in the 14 bilingual schools in the Zaolzie region of the Polish-Czech border. Although other studies suggest there may be three sociocultural competence components, our analysis suggests that they were integrated across two factors emphasizing intra- and inter-personal dimensions of sociocultural competence. Studies mainly from the United States describe whether and how students in dual language programs develop identities as bilingual and bicultural individuals, skills to interact across linguistic and cultural lines, and, more recently, awareness of dynamics between linguistic and cultural groups.

Based on this work, we developed an instrument to assess the extent to which bilingual schools in a specific border region attended to these cognitive, social, and political components of sociocultural competence. Our findings from the exploratory factor analysis revealed that a simple three-factor solution was not a good match to the data. Instead, the best model was a two-factor solution with the three components of sociocultural competence integrated. The first identified factor emphasized intrapersonal dimensions of sociocultural competence, and included cognitive, social, and political components of students’ individual development, while the second factor emphasized interpersonal dimensions and included social and political components.

### Items essential in individual components of cross-border competence

Upon examining the results, a clear first factor emerged encompassing items related to developing student’s individual skills, motivation, and cross-linguistic understandings. The items associated with this factor included, for example, “Develop students’ bilingualism and biliteracy,” “Support positive perceptions of students’ and families’ languages and cultures,” and “Support awareness of differences in language status in the border region.” As with these example items, the items constituting this construct emphasized development of intrapersonal facets of sociocultural competence, which included cognitive (i.e., bilingualism and biliteracy), social (i.e., perceptions of others’ cultures), and political (i.e., language status) components. We observed factor loadings between 0.49 and 0.78 for the eight items that comprised Factor One (see Table 4). The internal consistency of these items was moderately high with a Cronbach’s alpha of 0.79.

The first factor considers the development of students’ bilingual and bicultural identities in schools for the Polish minority in the Czech Republic, located along the Polish-Czech border. In this region, students encounter the Polish language not only among the Polish minority in the Czech Republic but also, for example, when crossing the Polish border, which happens very often, even daily. Shaping a student’s bilingual and bicultural identity requires that teachers have specific methodological competences. Although this factor is more related to the general essence of bilingual education than it is specific to the borderland’s territorial context, teachers’ competences enabling them to motivate students to learn borderland languages and cultures should result from their belief these skills will be useful in everyday life. The teacher should be able to communicate to students that bilingualism and biculturalism have a significant personal potential for people who identify with the border region and link their professional and personal futures with this region. Therefore, bilingual and bicultural education in the borderland should be promoted by highlighting the opportunity to use the acquired knowledge and skills in various areas of everyday life, depending on the needs, and with full awareness of differences between the cultures and traditions of the nations that jointly inhabit the borderland. Such education cannot be conducted in isolation from historical knowledge, traditions, and the area’s cultural heritage. As the next factor illustrates.

In contrast with the first factor focused on intrapersonal facets of sociocultural competence, the second factor included items related to developing students’ capacity to engage interpersonally with and to understand others in the region. For example, this factor included items such as, “Develop students’ ability to engage in various cross-border relations,” and “Promote respect for majorities and minorities equally.” Overall, the items constituting this factor emphasized the social and political dimensions of interpersonal communication in the border region. The five items associated with this factor loaded on this factor between 0.44 and 0.66 (see Table 4). The internal consistency of these items was high with a Cronbach’s alpha of 0.83.

Regarding the second factor, strengthening students’ sense of identity within the specific border region also involves developing empathy and respect for various cultural groups coexisting in this area, representing the majority and national or ethnic minorities. Building among students a positive emotional relationship with the border region they live in requires the teacher to have the competence to understand cross-border cooperation in the institutional dimension (e.g., cooperation between Polish and Czech schools) and the development of multidimensional cross-border relations (e.g., employer-employee, supplier-customer, and student-teacher relations, etc.). It is essential to show students the potential benefits of immersing in the borderland’s multicultural environment, where they can use their language skills and engage in professional and social activities on a daily basis, thanks to their understandings of the cultural and historical context of life in the borderland. The social and community-creating function of bilingual and bicultural education in the borderland can directly shape students’ skills, serving the use of the unique potential associated with living in this specific area. It requires developing such competences among teachers who should be open to the development of cross-border institutional cooperation and, in a broader context, cross-border relations that will allow students to anchor themselves in this environment better thanks to cross-border activities offered to them by the school. Therefore, developing teachers’ competences to conduct this type of education requires them to discover the socio-cultural, socio-linguistic, and socio-emotional dimensions of socio-political and binational education, considering the Polish and Czech contexts. Developing bilingual, bicultural, and binational education requires the teacher first to acquire specific knowledge about the borderland and the life of the national minority to properly expose this element in education through, for example, inclusive educational methods and the ability to focus students’ attention on this dimension of education. Competences to cooperate with the local communities (representatives of nations living on the borderland, including national minorities) and the students’ families, often mixed families with roots in both nations, are also extremely important. Engaging these two groups in bilingual and bicultural education definitely helps students embrace the borderland’s social environment, develop bilingual and bicultural interactions in the border environment, use borderland languages and cross-cultural skills to engage in cross-border initiatives, or meet the expectations arising from the economic and social need to learn from each other and together as the result of complex cross-border processes.

## Conclusions

These findings suggest that schools in the Zaolzie region attend to the development of students’ cross-border sociocultural competence through both an intra- and interpersonal lens, emphasizing individual students’ linguistic and cultural development as well as their capacity to appreciate and interact in their highly politicized cross-border context. The integration of the cognitive, social, and political components across these lenses suggests that educators in this region do not treat them as separate constructs but rather as related ideas that should be addressed in tandem. Further, while the political component (i.e., the development of critical consciousness) has been treated as a separate construct needing specific instructional attention in dual language programs [4, 45], perhaps educators serving Polish minorities in the Zaolzie region are exceedingly aware of how the political component shapes their daily lives given their unique cross-border context and thus infuse it across their programming.

In the Zaolzie region, not only are Polish-Czech cross-border relations important, but attention must also be paid to the existence of a Polish minority in the Czech part of the region. On the one hand, there is a need to maintain a certain balance in the relations between cooperating nations, but on the other hand, the existence of the Polish minority on the Czech side of the border creates an additional element in this relationship that, at the same time, is a testimony to the past armed conflicts and political tensions between the two countries. As such, strengthening students’ bilingualism and ability to communicate across cultures within the border region also involves developing empathy and respect for various cultural groups coexisting in the space, representing both the dominant group and national or ethnic minorities. Building strong bilingual and bicultural identities, as well as a positive emotional connection to the border region, requires that schools show students the potential benefits of immersing themselves in the border context where they can, on a daily basis, use their linguistic and intercultural communication skills to engage in professional and social activities that attend to the complex power dynamics they encounter.

Our study, though exploratory, has implications for both practice and research. With respect to the practical implications of this study, educators in the Zaolzie region can consider explicitly naming the intra- and interpersonal dimensions identified here as programmatic goals and assessing students’ progress in these two areas over time to measure program effectiveness. By calling out these dimensions of their school programming, educators in the region may also find it helpful to examine how their instructional planning and teaching practices align with each dimension and to use their findings in professional learning and program development activities.

In terms of implications for research, given that the researchers did not find evidence that bilingual/bicultural identity, cross-cultural competency, and critical consciousness were distinct constructs, but rather were integrated across intra- and interpersonal dimensions, more research is needed to understand if this integration was unique to cross-border sociocultural competence in the Zaolzie region or if the dimensions can be extended to the development of sociocultural competence in other contexts. Future research can use the items developed in this study to examine schools’ attention to sociocultural competence in other contexts and whether and how the context influences how the various components of sociocultural competence are addressed.
